# The Effects of Nudges: One-Shot Only? Exploring the Temporal Spillover Effects of a Default Nudge

**DOI:** 10.3389/fpsyg.2021.683262

**Published:** 2021-09-13

**Authors:** Merije Van Rookhuijzen, Emely De Vet, Marieke A. Adriaanse

**Affiliations:** ^1^Consumption and Healthy Lifestyles Group, Wageningen University and Research, Wageningen, Netherlands; ^2^Department of Social, Health and Organizational Psychology, Utrecht University, Utrecht, Netherlands

**Keywords:** nudge, choice architecture, default, temporal spillover, prosocial behaviour, food choice

## Abstract

Nudges, such as defaults, are generally found to be effective in guiding immediate behavioural decisions. However, little is known about whether the effect of a nudge can be lasting, meaning that it spills over to subsequent similar choices without the presence of a nudge. In three experiments, we explored the temporal spillover effects of a default nudge. The results of Experiments 1 (*N* = 1,077) and 2 (*N* = 1,036) suggest that nudging participants into completing a longer questionnaire affected their decision for the same behaviour a day later without the presence of a nudge. However, nudging participants into a healthier food choice in Experiment 3 (*N* = 969) did not result in such a temporal spillover effect. The results indicated that participants' change in attitude towards the nudged behaviour may partly explain the temporal spillover effects. These findings suggest that for some, but not all behaviours, default nudges may have the potential to yield temporal spillover effects and warrant a further investigation of boundary conditions and facilitators of the spillover effects of nudges.

Since Thaler and Sunstein's popular book “Nudge” (2008), the term “nudging” has become widely known and the concept had quite some impact in the area of public policy. Perhaps most notably, it inspired the establishment of numerous Behavioural Insights Teams (BITs) all over the globe, whose main aim is to inform and improve public services by generating and applying behavioural insights (Behavioural Insights Team, n.d.). This seems to be with good reason: Nudges are able to affect a wide range of behaviours (from increasing organ donation to improving healthy eating) while preserving the freedom of choice of individuals (Vecchio and Cavallo, 2019; Madden et al., 2020). Yet, surprisingly, the question of whether or not the effects of nudges are sustained over time has hitherto not received considerable attention. For some behaviours, such as opting to be an organ donor or choosing an energy provider, this may not be a particularly pressing question, since these are generally decisions that one does not or cannot revise often, and that can have a major impact in and of themselves. However, many nudge interventions actually target behaviours that people perform multiple times a day, such as physical activity, recycling, and eating. In this instance, influencing a single decision [e.g., nudging train travellers to take an apple at a snack shop (Kroese et al., 2016)] would probably have a rather limited impact on behaviours, such as general physical activity levels or overall healthy eating patterns. A more sustained behavioural change across time and contexts is generally required to make an impact on physical activity levels, recycling behaviour, or healthy eating patterns. For such behaviours, it is, therefore, worthwhile to explore whether nudges can also influence decisions beyond the nudged choice.

There is a large body of literature investigating the effects of behaviour on subsequent behaviour, so-called spillover effects (e.g., Guadagno et al., 2001; Mead et al., 2009; Sachdeva et al., 2009). Here, spillover effects are often defined as the effects of one behaviour on a second, *different* behaviour that occurs after the first behaviour (Dolan and Galizzi, 2015). We will label these types of spillover effects as *behavioural* spillover effects. The results of these studies suggest that behaviour can impact subsequent different behaviour in both a promoting and an inhibiting manner. However, little is known about the extent to which behavioural spillover effects also occur when a certain behaviour is the result of nudging. If nudged choices could have a positive impact on related but unnudged choices, this could dramatically increase the impact of nudges on overall and sustained behaviour change. This would, for example, be the case when nudging people into buying fruit would lead to an increase in choosing other healthy products. In addition to behavioural spillover effects, spillover effects can also be temporal in nature. Such spillover effects occur when a decision in a specific context is repeated in the same context at a later point in time. This would be the case when, for example, one has to choose between a light and regular soda drink in the supermarket, when the light option was previously successfully nudged.

Although a few studies have empirically investigated temporal spillover effects in the context of nudging, there are several established theories in psychology that do point to this possibility, such as self-perception theory (Bem, 1972) and self-herding (Ariely and Norton, 2008). That is, in both self-perception theory and the literature on self-herding, the central premise is that new attitudes and preferences are not just determinants of our behaviour, but can sometimes also be the product of behaviour as they can be formed by observing our own behaviour. This is especially true when we are unable to attribute our behaviour to an external source. Without such a source to justify behaviour then, internal attribution takes place, reasoning that it must have been a positive attitude towards the behaviour that caused it. This change in attitude may in turn affect subsequent decision-making in line with the changed attitudes, as has also been observed in research on cognitive dissonance (Festinger, 1962; Harmon-Jones and Mills, 2019). Considering that there is ample evidence demonstrating that people are frequently unaware of or underestimate the impact of nudges on their decisions (e.g., van Gestel et al., 2021), it is not unlikely that nudged choices are frequently misattributed to internal states. This study examines whether this indeed implies that nudges yield significant changes in attitudes and whether these changes are translated into subsequent temporal spillover effects.

We could even go a step further in our understanding of the possible positive spillover effects of nudges through internal attribution. That is, the nudged behaviour could even affect identity formation. The idea that behaviour can serve as the input to the formation of our identity stems from the work of Gneezy et al. (2012) and Bénabou and Tirole (2011). They argue that, without any external justification for our behaviour, we may attribute it to us being “the person that does that kind of things.” This identity formation can then serve as the input for later behaviours. To illustrate, Burger and Caldwell (2003) found that a change in self-concept (by asking participants to which extent they thought of themselves as persons who engage in various altruistic behaviours) could explain why participants were more likely to spend their time on voluntarily sorting and boxing canned goods if they were earlier asked to sign a homelessness petition. In the case of nudges, this would mean that the behaviour originally driven by a nudge could become internalised so that, in turn, this change in identity triggers similar behaviour once the nudge is no longer present. In contrast to a behaviour-specific attitude change, a change in identity implies that a possible spillover effect could even generalise to *different* but related domain-specific behaviours, and thus yield behavioural as well as temporal spillover effects. Next to these theoretical arguments why spillover effects could occur because of the misattribution of behaviour to positive attitudes or identity, spillover effects could also occur because of the desire to act consistently (Dolan and Galizzi, 2015): One simply behaves as one behaved previously, directly repeating the previous choices that are not mediated by attitudes or other relevant cognitions.

Although it can be argued that nudges could induce temporal spillovers based on the abovementioned theories, it should not be ignored that there is also the possibility of no spillover of the nudged behaviour to subsequent behaviour. More specifically, we assume that people are aware of the behaviour they perform following the nudge. It is this behaviour that could serve as the input for a changed attitude or identity. However, it is not necessarily the case that people are aware of their behaviour following the nudge. In that case, the default tendencies of people to behave in a particular way may be overridden by the nudge, but after removal, they will just resort to the decisions they would have made without the nudge. For example, by placing healthy snacks within the reach of a person would make him or her more likely to eat the snack without much thinking just because it is easy to grab. Intake of the health snack may seize when put back into the drawer that it came from, i.e., when the nudge is removed.

To date, research on nudging and spillover effects is relatively limited. Most studies examining nudge interventions solely consider their effects during the intervention itself. Once the nudge has been removed, data collection generally comes to a halt. However, in those few studies that did continue behavioural measurements after nudge removal, it is commonly found that the effect of the nudge indeed continues, albeit to a lesser extent than during the intervention. For example, Venema et al. (2018) used a default nudge to promote stand-up working for 2 weeks. The effects of the intervention were still noticeable even after 2 months, although they were not as strong as during the intervention. Although these results are promising, a major drawback of these studies is that data collection is almost solely based on group-level observations. Therefore, it cannot be concluded with certainty that the effects of nudges can persist on an individual level. Moreover, the study of responsible mechanisms using group level observations presents problems since the internal states of the individual cannot be coupled with their behaviour. To improve our understanding of whether, when, and how nudges may have the potential of spillover to subsequent decisions, it is thus important to systematically study the consequences of nudges on an individual level after their removal.

We know of only four studies (with a total of 11 experiments) specifically examining the spillover effects of nudges in the individual level. In these experiments, behaviour is measured following a nudge and is measured again after nudge removal. In four experiments, no default effects were found on the first measurement (Donkers et al., 2020, Experiments 1–3; d'Adda et al., 2017), making it impossible to conclude whether spillover effects follow effective nudges. In the experiments in which the nudge does influence the initial behaviour, no spillover effects (Ghesla et al., 2019; Kuhn et al., 2020, Experiments 1–3; Zimmermann and Renaud, 2021) or even compensating effects (Donkers et al., 2020, Experiment 4) are found. However, in all these experiments, the initial choice set differed from the subsequent choice set in one or more ways. In other words, all these studies investigated *behavioural* spillovers. For example, in the study of Ghesla et al. (2019), a dictator game was used to first nudge participants into donating money to charity, using either a weak or strong default nudge. They subsequently played another dictator game. However, this time they were not invited to donate money to charity but to another participant. While the aforementioned studies suggest that behavioural spillover effects of nudging may not be very likely, it is yet to be determined whether nudges may in fact lead to temporal spillover effects. Seeing that attitudes are more predictive the more specific they are to the behaviour that is predicted (Ajzen and Fishbein, 1977), any change in attitudes regarding a previously performed behaviour should therefore be particularly likely to affect the same behaviour in the same situation at a later time point. This would suggest that if attitudes are indeed affected by nudging interventions, temporal spillover effects are more likely to be observed than behavioural spillover effects.

Building on the aforementioned rationale, in the present paper we aimed to systematically explore potential temporal spillover effects of a default nudge in three preregistered experiments. We explicitly chose a default nudge since it is generally considered a prototypical System 1 nudge (Thaler and Sunstein, 2008; Hansen and Jespersen, 2013). Such nudges are thought to influence behaviour through System 1 processing, which is fast, automatic, and intuitive (Kahneman, 2011). We consider it as a prerequisite that people are unaware of the influence of the nudge on their behaviour for the occurrence of temporal spillover since only then can behaviour be misattributed to internal states and serve as an input for later behaviour.

In all three experiments, we measured the behaviour of participants on two consecutive days. Participants were randomly assigned to either a control condition or an experimental condition. On the 1st day, a default nudge was used to influence participants' behaviour in the experimental condition. On the 2nd day, the default nudge was removed and the behaviour was measured again. This setup allowed an examination of whether the effect of the nudge on the 1st day continued to the 2nd day when it was no longer present. In Experiment 1, we tested the temporal spillover effect of a default nudge on prosocial behaviour, by asking participants whether they opted for completing a longer version of a questionnaire that would take five additional min without getting any extra reimbursement. In Experiment 2, we aimed to replicate and extend the findings from Experiment 1 and used a similar design to explore possible changes in the attitude of the participants and their identities as mechanisms responsible for the temporal spillover effect. In Experiment 3, we tested whether the results of Experiments 1 and 2 could be replicated with food choices, by asking participants to choose between unhealthy food products and healthier alternatives.

## Experiment 1

In Experiment 1 (preregistered at the Open Science Framework: https://osf.io/s2f3j), we aimed to investigate whether the effect of a nudge continues once the nudge is removed. To this end, we asked participants on two consecutive days whether they opted for completing a longer version of a questionnaire that would take 5 min more without getting extra reimbursement (Wachner et al., 2020 based on Paunov et al., 2020). Participants were randomly assigned to an experimental condition or a control condition. On the 1st day, participants in the experimental condition were nudged into completing the longer questionnaire by preselecting the option (a default nudge). No nudge was used in the control condition. On the 2nd day, the nudge in the experimental condition was removed. Building on the theories that predict that behaviour can also be seen as the input to affect internal states (e.g., attitudes and identities), which, in turn, affect subsequent behaviour (Bem, 1972; Ariely and Norton, 2008; Bénabou and Tirole, 2011; Gneezy et al., 2012), we expected to find (1) an effect of the nudge on questionnaire choice on the 1st day and (2) an effect of the initial nudge on questionnaire choice on the 2nd day even when the nudge was no longer present.

### Method

#### Participants

Participants were recruited *via* the online crowdsourcing website *Prolific Academic*. Participants could only participate when they were aged 18 years or older, spoke English fluently, had two or more previous submissions on Prolific Academic, and had a 95% or more approval rate on Prolific Academic. These last two criteria were added to minimise the attrition rate. Participants were encouraged to participate on a desktop and rewarded with £2.00.

A sample size calculation with the software program G^*^Power 3.1.9.2 (Faul et al., 2007) resulted in a recommended sample size of 263 (with 0.90 power and small to medium effect size of ϕ = 0.2). Because we expected some dropout, we recruited 50% extra participants on day 1 of the experiment, resulting in 395 participants on the 1st day (of which 358 also participated on day 2).

Initial analyses of the effectiveness of the nudge manipulation on questionnaire choice on day 1 of this data showed a clear trend for the expected effect of the manipulation, with 57.9% of participants in the experimental condition choosing the longer version of the questionnaire vs. 51.4% in the control condition. This signalled that the effect of the default on day 1 may have been smaller than expected (ϕ = 0.065), which may have caused the effect of the manipulation to be non-significant. As an effect of the manipulation on day 1 needed to be detected before any possible temporal spillover effects on day 2 could be assessed, we decided to recruit more participants to be able to detect a small effect on day 1. A sample size calculation (with 0.90 power and small effect size of ϕ = 0.1) resulted in a recommendation of 1,051 participants, which meant an addition of 693 participants. Assuming a dropout rate similar to that of the first data wave (90.6%), 765 extra participants were needed on day 1.^1^

Combining the data of the two waves, a total of 1,163 participants finished the questionnaire on day 1, of which 1,077 also finished the questionnaire on day 2 (92.6% response rate). Of these 1,077 participants, 533 (49.5%) had been randomly allocated to the experimental condition. The average age was 32.56 years (*SD* = 10.48), with 50.0% men, 49.8% women, and 0.3% indicating “other.” The highest completed level of education was a high-school diploma with 34.7% and a bachelor's degree with 40.0% of participants. Participants had 55 different nationalities with most participants coming from the UK (33.5%) and Poland (14.2%).

#### Design

The experiment was conducted with a 2 (between-subjects factor = Condition: experimental vs. control) × 2 (within-subject factor = Day: 1 vs. 2) mixed design with the questionnaire choice (normal/longer) as the dependent variable.

#### Procedure

On day 1, participants were invited to complete a questionnaire about lifestyle, as part of our cover storey. They were told that, based on their answers, they might be invited for another study that would be conducted the next day. Participants were kindly requested only to participate in the present study if they felt that they could also complete the second study the next day. After giving their informed consent, participants were asked about some demographics. Half of the participants were then nudged into filling in a longer questionnaire as this would help improving future questionnaires without getting any extra reimbursement. After selecting a questionnaire, participants completed a normal or longer bogus questionnaire in line with our cover story to make participants actually perform the behaviour.

On the 2nd day, only participants who completed the questionnaire on day 1 were invited for another study on lifestyle. Participants first had to give their informed consent and were asked about some demographic variables. Participants were then asked to choose a normal or longer version of a questionnaire about lifestyle. None of the participants were nudged on this day. As before, a bogus questionnaire, which corresponded in length to the selected version (long or normal), was completed. However, the questions differed from the questions on the 1st day.^2^ Participants were then thanked and debriefed about the real aim of the study.

### Measures and Materials

#### Demographics

Participants were asked for their age (in years), gender (male, female, or other), nationality (from a dropdown list of 193 nationalities), and the highest degree of completed educational level [less than a high school diploma, high school degree or equivalent, bachelor's degree, master's degree, doctorate, or other educational levels (please specify)].

#### Manipulation

Using a previously tested nudge manipulation (Wachner et al., 2020 based on Paunov et al., 2020), all participants were asked the following question on day 1: “Please indicate whether you will participate in the long version of this study (12 min) or normal version (7 min). If you choose to participate in the long version, you will not receive additional payment; however, you will help to improve future questionnaires.” Participants were randomly assigned to the experimental or control condition. In the experimental condition, the option of completing the longer version was preselected on day 1 (a default nudge). No option was preselected in the control group. On day 2, the same question was asked, but this time, both conditions received the question without any nudge (no specific option was preselected). We chose this manipulation because the decision made by participants to complete the normal or longer questionnaire was not a hypothetical one, but was real and actually impacted the amount of time that participants spent on the questionnaire (the behaviour of interest in this study). Moreover, the manipulation is credible, since being asked to complete two studies on two consecutive days and to complete a longer version of these questionnaires without getting any extra reimbursement would not immediately raise suspicion.

#### Bogus Questionnaire

On both days, (parts of) existing questionnaires or made-up items in the domains of personality, lifestyle, and eating behaviour were used. Fewer items were used in the normal version than in the longer version.

#### Data Preparation

Being preregistered, participants were only included when they finished both questionnaires. If answers to demographic questions differed between the 2 days (e.g., two different nationalities), participants were contacted for the right information. Cells with less than five observations were set missing (as was the case with gender) or merged (as was the case with nationality) to make the variable viable for inclusion in the analyses. When participants indicated “other” as their education, their specification was transformed into one of the listed options by the researcher. When outliers (three SDs more than or less than the mean) were detected within variables for a particular analysis, these values were set missing.

### Results

#### Confirmatory Analyses

##### Randomisation Check

We first examined whether the demographic variables were equally distributed across the control and experimental condition using an individual *t*-test and a chi-square tests. No differences were found regarding age [*t*(1,065) = 0.266, *p* = 0.791], gender [χ^2^(1) = 0.301, *p* = 0.583], nationality [χ^2^(13) = 19.957, *p* = 0.096], and the level of education [χ^2^(4) = 0.543, *p* = 0.969], indicating successful randomisation.

##### Manipulation

To test whether the nudge had the intended effect on day 1, a chi-squared test was conducted with condition (experimental/control) as the independent variable and questionnaire choice day 1 (QC1) (normal/longer) as the dependent variable. As expected, significantly more participants choose the longer version of the questionnaire in the experimental condition (57.2%, 95% CI [53.0, 61.4]) than in the control condition (50.9%, 95% CI [46.7, 55.1]) [χ^2^(1) = 4.308, *p* = 0.038]. This means that the manipulation was successful although the effect was small (ϕ = 0.063).

##### Temporal Spillover Effect

**Figure 1:**
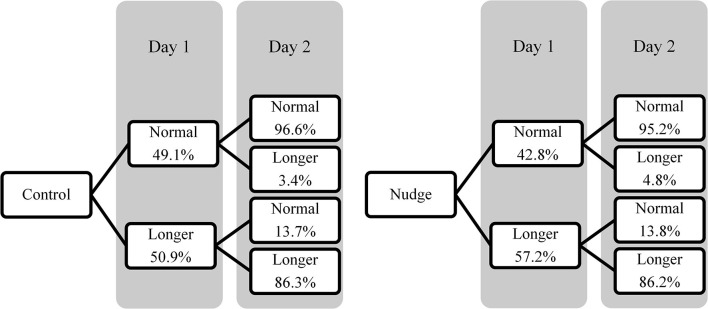
Percentages of participants choosing the normal and longer version of the questionnaire on days 1 and 2 in Experiment 1.

To test for a temporal spillover effect, a chi-squared test was conducted with condition (experimental/control) as the independent variable and questionnaire choice on day 2 (QC2) (normal/longer) as the dependent variable. Figure 1 shows the percentages of participants choosing the normal or longer version on day 2 relative to their choice on the 1st day in the control and experimental condition. A trend was observed in which more participants in the experimental condition (51.4%, 95% CI [47.2, 55.7]) chose the longer version than in the control condition (45.6%, 95% CI [41.4, 49.8]) [χ^2^(1) = 3.650, *p* = 0.056]. However, this effect was small (ϕ = 0.058).

#### Exploratory Analyses

##### Mediation of Condition on QC2 Through QC1

For exploratory purposes, we also assessed whether the nudge had an indirect effect on QC2 through its initial effect on QC1. Therefore, we conducted a mediation analysis according to the steps described in Iacobucci (2012) to test whether QC1 (normal/longer) mediated the effect of condition (experimental/control) on QC2 (normal/longer). The mediation analysis resulted in a significant *Z*-mediation value of 2.051 (*p* = 0.040). This means that the nudge influenced QC1, which, in turn, influenced QC2.

### Discussion

In Experiment 1, a trend in which the effect of the default nudge on prosocial behaviour spilled over to subsequent similar behaviour was observed. However, this effect was only small, which may partly be attributed to the small effect of the nudge on day 1. A significant mediation of QC1 on the effect of condition on QC2 was found, suggesting that the effect of the nudge on the 1st day may be seen as a prerequisite for its continued effect on the 2nd day once the nudge is removed.

As discussed in the introduction, several theories predict that an attitude (Bem, 1972; Ariely and Norton, 2008) or even identity (Bénabou and Tirole, 2011; Gneezy et al., 2012) change could explain the temporal spillover effect. Therefore, in Experiment 2, we examined the options of a mediated pathway of changed attitudes and changed identity between two similar behaviours of which the first was initially elicited by the nudge.

## Experiment 2

**Figure 2:**
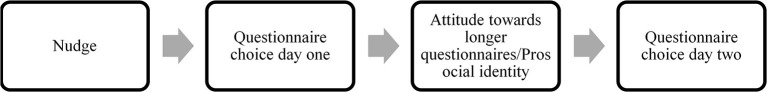
Hypothesised pathways for Experiment 2.

To substantiate the trend observed in Experiment 1 for a temporal spillover effect, we replicated the manipulation in Experiment 2 (preregistered at AsPredicted: Preregistration 37220). Moreover, we examined a change in attitude towards the initial behaviour and a change in prosocial identity as possible mediators explaining the relation between the nudge and the behaviour on the 2nd day. We used a similar setup as in Experiment 1 while adding participants' attitude towards taking longer questionnaires and their prosocial identity as possible mediators. As in Experiment 1, we expected to find (1) an effect of the nudge on QC1, (2) an effect of the nudge on QC2, (3) a mediation effect of condition on QC2 through QC1, and (4) a mediating effect QC1 on QC2 through participants' attitude towards taking longer questionnaires and/or prosocial identity (Figure 2 is a summary of the hypotheses).^3^

### Methods

#### Participants and Design

Participant recruitment and inclusion criteria were similar to Experiment 1. A sample size calculation using the software program G^*^Power 3.1.9.2. resulted in a recommended sample size of 1,051 (with 0.90 power and a small effect size of ϕ = 0.1) for the effect of the nudge on days 1 and 2. Taking into account the dropout rate of Experiment 1 (6.90%), 1,150 participants were recruited on day 1.

On day 1, 1,150 participants finished the questionnaire, of which 1,044 also finished the questionnaire on day 2 (90.8% response rate). Of these, eight participants were excluded since they correctly guessed the aim of the study. Therefore, analyses were conducted on 1,036 participants. Of these participants, 512 (49.4%) had been randomly allocated to the experimental condition. The average age was 29.01 years (SD = 8.55), with 53.9% men, 45.9% women, and 0.2% indicating “other.” The highest level of education completed by the participants was a high school diploma in 36.6% and a bachelor's degree in 36.6% of participants. Participants had 64 different nationalities with most participants coming from the UK (22.7%), Poland (13.5%), and Portugal (12.1%).

#### Design

The design of Experiment 2 was similar to Experiment 1 with the addition of two possible mediators: participants' attitude towards taking longer questionnaires and their prosocial identity.

#### Procedure

The procedure of Experiment 2 was similar to that of Experiment 1. However, after the manipulation on day 1, participants' attitude towards taking longer questionnaires and their prosocial identity were additionally measured. The order of these two variables was counterbalanced. On day 2, these variables were measured again.^4^ At the end of the 2nd day, participants were asked about the goal of the study.

### Measures and Materials

#### Manipulation

The manipulation in Experiment 2 was similar to that of Experiment 1. However, instructions were slightly changed to highlight the prosocial element of the choice. The instructions now read: “Please indicate whether you will participate in the longer version of this study (12 min) or normal version (7 min). If you choose to participate in the longer version, you will not receive additional payment; however, you will help researchers in improving their future questionnaires.” This wording made it more apparent that taking the longer version would help others and allowed for matching our attitude measure to the nudged behaviour. Since the duration of both questionnaires was shorter than expected in Experiment 1, we added the extra questions to the questionnaire measuring attitude and identity instead of replacing questions.

#### Attitude Towards Taking Longer Questionnaires

The attitude of participants towards taking longer questionnaires was measured by taking the mean of six items on a seven-point semantic differential scale (as in Aertsens et al., 2011). Participants were presented with the sentence “Filling in the longer questionnaire is…” followed by sliders with various anchors (good/bad, positive/negative, satisfying/unsatisfying, enjoyable/unenjoyable, pleasant/unpleasant, and preferable/unpreferable). Cronbach's alpha (α = 0.894) was deemed high enough to average the items into one scale.

#### Prosocial Identity

Participants' prosocial identity was measured by taking the mean of two items: “To what extent do you see yourself as a helpful person” and “To what extent do you see yourself as an unselfish person,” to which participants had to rate themselves on a seven-point Likert scale ranging from (1) not at all to (7) a great extent. The two items showed a moderately positive correlation [*r*(1) = 0.458, *p* < 0.001], which for our purposes was deemed high enough to continue with the items as one scale. To mask the goal of the study, several filler items were added between the items of interest.

#### Data Preparation

Data preparation in Experiment 2 was similar to that in Experiment 1.

### Results

#### Confirmatory Analyses

##### Randomisation Check

The randomisation check using an individual *t*-test and a chi-squared tests showed no difference between the control and experimental condition regarding gender [χ^2^(1) = 0.353, *p* = 0.552], nationality [χ^2^(14) = 9.135, *p* = 0.822], and level of education [χ^2^(4) = 1.675, *p* = 0.795]. However, participants in the experimental condition (*M* = 29.82, SD = 9.19) were significantly [*t*(1,022) = 2.983, *p* = 0.003, *d* = 0.186] older than participants in the control condition (*M* = 28.23, SD = 7.80). Therefore, all analyses were also run with age added as a covariate. Since this did not change the results, the final analyses are reported without the addition of age as a covariate.

##### Manipulation

To test whether the nudge had the intended effect on day 1, a chi-squared test was conducted with condition (experimental/control) as the independent variable and QC1 (normal/longer) as the dependent variable. As expected, significantly more participants chose the longer version of the questionnaire in the experimental (64.3%, 95% CI [60.1, 68.4]) than in the control (55.5%, 95% CI [51.3, 59.8]) condition [χ^2^(1) = 8.216, *p* = 0.004]. This means that the manipulation was successful although the effect was small (ϕ = 0.089).

##### Temporal Spillover Effect

**Figure 3:**
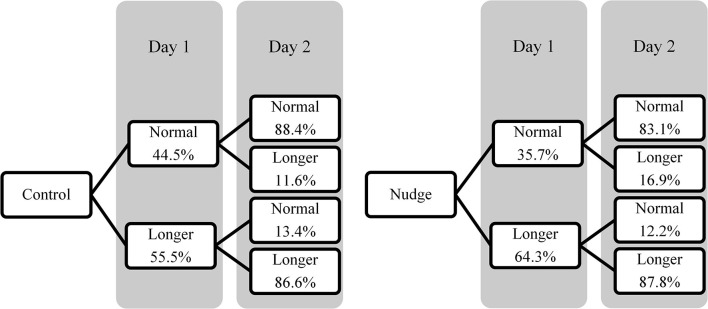
Percentages of participants choosing the normal and longer version of the questionnaire on days 1 and 2 in Experiment 2.

To test for a temporal spillover effect of the nudge on the behaviour after nudge removal, a chi-squared test was conducted with condition (experimental/control) as the independent variable and QC2 (normal/longer) as the dependent variable. Figure 3 shows the percentages of participants choosing the normal or longer version on day 2 relative to their choice on the 1st day in the control and experimental condition. Although the effect was small (ϕ = 0.094), significantly more participants chose the long version of the questionnaire in the experimental condition (62.5%, 95% CI [58.3, 66.7]) than in the control condition (53.2%, 95% CI [49.0, 57.5]) (χ^2^ = 9.096, *p* = 0.003). This means that there was a temporal spillover of the nudge on QC2.

#### Exploratory Analyses

##### Mediation of Condition on QC2 Through QC1

To examine a possible mediation effect of QC1, a mediation analysis according to the steps described in Iacobucci (2012) was conducted to test whether QC1 (normal/longer) mediated the effect of condition (experimental/control) on QC2 (normal/longer). The mediation analysis resulted in a significant *Z*-mediation value of 2.834 (*p* = 0.005). This means that the nudge influenced QC1, which, in turn, influenced QC2.

##### Mediation of QC1 on QC2 Through Attitude Towards Taking Longer Questionnaires and Prosocial Identity

Participants choosing the longer questionnaire on day 1 had a significantly more favourable attitude towards taking longer questionnaires (*M* = 5.06, SD = 1.15) than participants choosing the normal questionnaire on day 1 (*M* = 3.86, SD = 1.03) [*t*(1,034) = −17.213, *p* < 0.001, *d* = 1.091]. Moreover, participants choosing the longer questionnaire on day 1 also had a significantly higher prosocial identity (*M* = 5.11, SD = 0.91) than participants choosing the normal version on day 1 (*M* = 4.94, SD = 1.00) [*t*(1,025) = −2.881, *p* = 0.004, *d* = 0.183]. However, the experimental and control condition did not differ significantly regarding their attitude towards taking longer questionnaires [*t*(1,034) =-0.065, *p* = 0.948] and their prosocial identity [*t*(1,025) = 1.149, *p* = 0.251].

A mediation analysis was conducted with the PROCESS macro for SPSS (model 4) using a 95 percentile bootstrap approach with 5,000 samples to test whether the attitude of the participants towards taking longer questionnaires and their prosocial identity mediated the effect of QC1 on QC2 (Hayes, 2017). The mediation analysis indicated a significant indirect effect of participants' attitude towards taking longer questionnaires (*B* = 0.4930, SE = 0.1067, 95% CI [0.2968, 0.7144]), but not for the prosocial identity of the participants (*B* = −0.0059, SE = 0.0180, 95% CI [0.0423, 0.0323]). This means that only participants' attitude towards taking longer questionnaires, and not their prosocial identity, mediated the effect of QC1 on QC2.^5^

### Discussion

In Experiment 2, the results suggested a small temporal spillover effect of the nudge on QC2 and also a mediation effect of the nudge on QC2 through QC1. Moreover, participants' attitude towards taking longer questionnaires mediated the effect of QC1 on QC2. These findings suggest that the nudge affected the behaviour on day 1, which affected participants' attitude towards the behaviour, which, in turn, affected the behaviour on day 2. No mediating role of the prosocial identity of the participants was found. In Experiment 3, we aimed to see whether a temporal spillover effect could also be present in another domain in which choices are made on a daily basis: eating behaviour.

## Experiment 3

In Experiment 3 (preregistered at As Predicted: 41062), we aimed to test whether a temporal spillover effect could be observed by using food choice as our behaviour of interest, using the manipulation of van Gestel et al. (2021). To this end, participants had to make choices in an online supermarket. Participants were randomly assigned to an experimental or control condition. In the experimental condition, participants were nudged into choosing the healthier option on the 1st day using a default nudge. Similar to the previous experiments, we expected to find (1) an effect of the nudge on the proportion of nudged food choices on day 1 (NFC1), (2) an effect of the nudge on the proportion of nudged food choices on day 2 (NFC2), (3) a mediation effect of the nudge on the proportion of nudged food choices on NFC2 through NFC1, and (4) a mediation effect of NFC1 on NFC2 through participants' attitude towards choosing healthier food products.

### Methods

#### Participants and Design

Participant recruitment and inclusion criteria were identical to those used in Experiment 1, with the exception that only people from the UK could participate, because some food stimuli are only available in the UK. A sample size calculation with the software program G^*^Power 3.1.9.2. resulted in a recommended sample size of 1,052 (with 0.90 power and a small effect size of *d* = 0.2). Averaging the dropout rate (92.125%) of Studies 1 and 2 lead to a necessary recruitment of 1,142 participants on day 1. Participants were rewarded with £1.00.

On day 1, 1,139 participants finished the study, of which 982 also finished the study on day 2 (86.2% response rate). Of these, 13 participants were excluded since they correctly guessed the aim of the study. Therefore, analyses were conducted on 969 participants. Of these participants, 485 (50.1%) had been randomly allocated to the experimental condition. The average age was 38.76 years (SD = 13.54), with 36.8% men, 63.0% women, and 0.2% indicating “other.” The highest level of education completed by the participants was a high school diploma in 36.6% and a bachelor's degree in 39.3%.

The experiment was conducted with a 2 (between-subjects factor = Condition: experimental vs. control) × 2 (within-subject factor = Day: 1 vs. 2) mixed design with the proportion of healthier food choices that were nudged on the 1st day (varying from 1 to 10) as the dependent variable. The attitude of the participants towards choosing healthier food products was added as a possible mediator.

#### Procedure

The procedure of Experiment 3 was similar to that of Experiment 2, with some minor alterations. Instead of asking participants whether they were willing to fill in a longer questionnaire, participants had to make 10 hypothetical food choices. Every choice set consisted of four options with two unhealthy products and two healthier alternatives. In the experimental condition, one of the healthier products was nudged by preselecting it and making it more salient. Moreover, the identity measure was removed from the questionnaire since it was found not to mediate the effect in Experiment 2. Finally, at the end of the 2nd day, participants were asked what they thought about the goal of this study.

### Measures and Materials

#### Demographics

In addition to measuring age, gender, and level of education as in Experiments 1 and 2, participants were now also asked how many hours and minutes ago they last ate and drank something. The answers were transformed into total numbers of minutes.

#### Manipulation

We used a task originally developed by van Gestel et al. (2021). Participants were presented with 14 food choice sets in an online supermarket environment. All 14 food choice sets consisted of four options that could be chosen by the participant. Ten of these choice sets consisted of two unhealthy products and two healthier alternatives. Four of these choice sets consisted of solely unhealthy or healthier food products. These were only incorporated as filler choices. Participants were given the instructions to choose the product of their liking and to put it in their online grocery basket. Participants were randomly assigned to the experimental or control condition. In the experimental condition, one healthier product was preselected by putting a border around it and made salient by enlargement (an example trial of the task can be found in van Gestel et al., 2021).

Since every (non-filler) trial consisted of two unhealthy and two healthier options and because only one healthier product was nudged, the chosen product to be nudged was randomised between the participants. The corresponding healthier food item in the control condition was always placed at the top left of the screen.

#### Attitude Towards Choosing Healthier Food Products

Participants' attitude towards choosing healthy food products was measured with a semantic differential scale (as in Aertsens et al., 2011). The scale started with the sentence “Choosing healthier food products is…” followed by seven-point Likert scales with various anchors (good/bad, positive/negative, satisfying/unsatisfying, enjoyable/unenjoyable, pleasant/unpleasant, and preferable/unpreferable). Participants had to provide answers on visual analogue scales ranging from 0 to 100. Cronbach's alpha (α = 0.856) was deemed high enough to average the items into one scale.

#### Data Preparation

Data preparation in this experiment was similar to that in Experiment 2.

### Results

#### Confirmatory Analyses

##### Randomisation Check

A randomisation check showed no differences between the control and experimental condition regarding age [*t*(966) = −1.340, *p* = 0.180], gender [χ^2^(1) = 0.390, *p* = 0.532], level of education [χ^2^(4) = 3.029, *p* = 0.553], minutes after eating on day 1 [*t*(967) = 0.221, *p* = 0.825], and minutes after eating on day 2 [*t*(942) = 0.325, *p* = 0.745], indicating a successful randomisation.

##### Manipulation

To test whether the nudge had the intended effect on day 1, a *t*-test was conducted with condition (experimental/control) as the independent variable and NFC1 as the dependent variable. As expected, NFC1 was significantly [*t*(963) = −5.709, *p* < 0.001] higher in the experimental (29.4%, 95% CI [27.6, 31.2]) than in the control (22.7%, 95% CI [21.2, 24.1]) group. This means that the manipulation was successful although the effect was small (*d* = 0.366).

##### Temporal Spillover Effect

To test for a temporal spillover effect of the nudge on the behaviour after the removal of the nudge, a *t*-test was conducted with group (experimental/control) as the independent variable and NFC2 as dependent variable. NFC2 was larger in the experimental condition (25.5%, 95% CI [23.8, 27.1]) than in the control condition (23.5%, 95% CI [22.0, 25.1]). However, this difference was not significant [*t*(961) = −1.680, *p* = 0.093]. This means that there was no temporal spillover effect of the nudge on NFC2.^6^

##### Mediation of Condition on NFC2 Through NFC1 and Attitude Towards Choosing Healthier Food Products

Participants with a higher NFC1 than expected based on chance (>0.25) had a significantly more favourable attitude towards choosing healthier food products (*M* = 76.40, SD = 15.81) than participants with a lower NFC1 than expected based on chance (<0.25) *(M* = 63.97, SD = 17.05) [*t*(961) = 11.674, *p* < 0.001, *d* = 0.756]. However, the experimental and control condition did not differ significantly regarding their attitude towards choosing healthier food products [*t*(965) = 0.017, *p* = 0.987].

To examine a possible mediation effect of condition on NFC2 through NFC1 and the attitude of the participants towards choosing healthier food products sequentially, a mediation analysis was conducted with the PROCESS macro for SPSS (model 6) using a 95 percentile bootstrap approach with 5,000 samples (Hayes, 2017). The mediation analysis indicated a significant serial mediation effect of NFC1 and the attitude of the participants towards choosing healthier food products for the effect of condition on NFC2 (*B* = 0.0017, SE = 0.0007, 95% CI [0.0006, 0.0032]). This means that the nudge influenced NFC1, which, in turn, influenced the attitude of the participants towards choosing healthier food products, which, in turn, influenced NFC2.^7^

### Discussion

In contrast to Experiments 1 and 2, the results of Experiment 3 did not point towards a temporal spillover effect of the nudge on food choice after its removal. However, as in Experiment 2, we found a chain of mediation in which the nudge affected food choice on the 1st day, which in turn affected the attitude of the participants towards choosing healthier food products, which in turn affected the food choice after removal on the 2nd day. This means that the nudge was not able to impact food choice after its removal directly, but it does provide preliminary evidence for behaviour to influence attitudes, as predicted by self-perception theory (Bem, 1972).

## General Discussion

In the current paper, three experiments were conducted to systematically, and on an individual level, explore the effect of nudges on subsequent similar choices once they have been removed, i.e., their potential temporal spillover effect. In Experiments 1 and 2, the results seemed to point towards a small temporal spillover of the default nudge promoting prosocial behaviour. However, in Experiment 3, no temporal spillover effect was found for a default nudge promoting hypothetical healthier food choices. In all three experiments, the initial choice predicted the subsequent non-nudged choice. Moreover, in Experiments 2 and 3, participants' attitude towards the nudged behaviour was measured and found to mediate the effect of the initial behaviour on the behaviour after the removal of the nudge.

These results seem to suggest that a single encounter with a nudge can affect subsequent similar behaviour after the removal of the nudge. More specifically, the mediating effect of the initial behaviour indicated that nudges are able to *prolong* their effects. This implies that it is a prerequisite of the nudge to affect the initial behaviour for it to continue after removal. However, these small temporal spillover effects do not occur for all behaviours as they were only found for prosocial behaviour and not for food choice. Future research is needed to see whether these mixed findings are indeed related to the behavioural domain or whether there are other reasons for this inconsistency.

That is, the experiments in which the small temporal spillover effect was found (Experiments 1 and 2) differed from the experiment in which no temporal spillover effect was found (Experiment 3) beyond the difference in the behavioural domain (eating or prosocial). For example, in Experiment 3, participants had to make multiple choices consisting of four options every day. All these choices were nudged on the 1st day in the experimental condition. In Experiments 1 and 2, participants only had to make one choice consisting of two options per day. Additionally, in Experiments 1 and 2, the choice participants made had actual immediate behavioural implications, which was not the case in Experiment 3. Each of these, and other differences, including the difference in behavioural domain, may have affected the potential of the nudge spilling over to a subsequent decision. Future research is therefore required not only to replicate the findings of Experiments 2 and 3 but also to systematically explore these and other factors facilitating or hindering the potential of temporal spillover effects.

Regarding the hypothesis that a change in attitude may be one of the processes responsible for the temporal spillover effect, we found that the effect of the actual choice (which was influenced by the nudge) on day 1 on the behaviour on day 2 was mediated by attitude towards the initial behaviour. In fact, although no temporal spillover effect was found, Experiment 3 showed some evidence for a sequential mediation model in which the nudge influenced the behaviour on day 1, which influenced attitude towards the behaviour, which, in turn, affected the behaviour on day 2. These results are in line with predictions from the self-perception theory of Bem (1972), which states that behaviour may follow attitudes but that behaviour can also be an input and a source for the formation of attitudes. In the current studies, participants were arguably not aware that an external stimulus, i.e., the nudge, affected their behaviour. To explain their own behaviour, they assigned it to a favourable attitude towards the behaviour, which, in turn, led them to continue the behaviour once the initial trigger (the nudge) was removed. Note that it is thus not the manipulation itself, but acting in line with this manipulation which is setting this attitude change and corresponding second choice in motion.

No evidence was found for the mediating role of participants' identity on the temporal spillover effect in Experiment 2. This may also explain why no spillover effects were found in previous studies (Ghesla et al., 2019; Kuhn et al., 2020), in which the behaviour was initially targeted by the nudge differed from the spillover behaviour (i.e., targeting *behaviour* instead of temporal spillovers) as such behavioural spillover effects would require attribution to more global cognitions affecting a broader range of behaviours. It should be noted that although an identity change did not mediate the temporal spillover effect of nudged prosocial behaviour in Experiment 2, it could have mediated the temporal spillover effect in Experiment 3. However, we did not measure healthy eating identity in Experiment 3. Therefore, we cannot yet draw a definitive conclusion about the role of identity in the temporal spillover effect.

## Limitations and Suggestions for Future Research

Some limitations of the current study should be noted. First of all, we only found temporal spillover effects in the behavioural domain of prosocial behaviour and not on food choice (although the sequence of events was similar to that of prosocial behaviour). Future research is needed to test whether the temporal spillover effect may be more pronounced in certain behavioural domains than others or whether the discrepancy between the experiments lies in the methodical differences like the multiple and hypothetical choices participants had to make in Experiment 3.

Second, the effect sizes of both the nudge manipulation and the temporal spillover effect were only small. In Experiment 1, the unexpected small effect size of the nudge made it necessary to include another wave of participants for the power to be high enough to detect such small effect sizes. In addition, the observed temporal spillover effect was only marginally significant, so the results of this study should be interpreted with care. Although the results of Experiment 1 were replicated and found to be significant in Experiment 2, still both the initial effect of the nudge as well as the temporal spillover effect were small. We should be aware of the fact that the nudge manipulation needs to work for temporal spillover effects to occur and that small nudge effects are likely to yield small temporal spillover effects. However, the non-invasive nature of nudges does imply that they can be implemented on a large scale, which means that even small effects can impact behaviour on a population level.

Third, as attitudes were not measured at baseline, it cannot be ruled out that participants already had formed attitudes towards taking longer questionnaires before participation and therefore consistently chose the normal or longer version on both days. While this is theoretically possible, this alternative explanation seems unlikely in view of the novelty of the target behaviour in the two studies in which we observed a temporal spillover effect (Experiments 1 and 2). That is, in these studies we explicitly chose to target a relatively unfamiliar choice—between a normal and a longer version of a questionnaire—for most participants, for which they likely did not have *a priori* attitudes. In addition, participants were randomly assigned to a nudge or control condition, which affected their attitudes and subsequent behaviour, making it unlikely that baseline differences in attitudes are responsible for the observed effects.

Fourth, while the present study focused on attitude change as the mechanism driving the temporal spillover effect, other potential mechanisms explaining the temporal spillover effect should not be ruled out. As mentioned in the introduction, one such mechanism may be the desire to act consistently (Dolan and Galizzi, 2015). Andrade and Ariely (2009) argue that such direct behavioural consistency is especially likely when contexts are highly similar, such as in the present studies where we investigated temporal spillover effects. Future research should be conducted to investigate the possibility and relative share of these processes in temporal spillover effects.

Fifth, when interpreting the findings of the present studies, it is also important to realise that we used only one type of nudge: a default nudge. Whether or not the potential of nudges to yield temporal spillover effects is restricted to defaults as a specific type of nudge remains unclear and should be explored in future research.

Finally, we only showed the temporal spillover effect for similar choices in similar contexts after 1 day. Future research should investigate whether spillover effects generalise to other behaviours and/or contexts and whether conclusions can also be generalised to behaviours with increasing time gaps.

## Conclusion

Notwithstanding these limitations, the present findings are among the first to show that default nudges can influence later choice decisions without nudges even after a single exposure, with the initial behaviour serving as an input for the later behaviour through an altered attitude. This result is a promising first step in the assessment of the extended influence of nudges although replication of these findings is warranted. Future research is also needed to examine this effect in the case of other behaviours and in other (real-life) situations.

## Data Availability Statement

The raw data supporting the conclusions of this article will be made available by the authors, without undue reservation.

## Ethics Statement

The studies involving human participants were reviewed and approved by the Social Sciences Ethics Committee Wageningen UR. The patients/participants provided their written informed consent to participate in this study.

## Author Contributions

MV collected and analysed the data and drafted the manuscript. All authors designed the experiments, reviewed, revised the manuscript, read, and approved the final manuscript.

## Conflict of Interest

The authors declare that the research was conducted in the absence of any commercial or financial relationships that could be construed as a potential conflict of interest.

## Publisher's Note

All claims expressed in this article are solely those of the authors and do not necessarily represent those of their affiliated organizations, or those of the publisher, the editors and the reviewers. Any product that may be evaluated in this article, or claim that may be made by its manufacturer, is not guaranteed or endorsed by the publisher.

## Footnotes

^1^We are aware that adding extra participants in a second wave of data collection after initial analyses is not recommended, and could be seen as p-hacking. We do not take this threat to the reliability of our findings lightly, which is why we are transparent about this and urge readers to be careful in interpreting the findings of the present study. However, we also think that our decision is justified as we only looked at an effect of the nudge on the 1st day and did not run any analyses on the hypothesised temporal spillover effect. In order to test the potential of a temporal spillover effect, it is a prerequisite to be able to detect an initial effect of the nudge. Therefore, we argue that the extra recruitment is justified in this case to allow for a sufficiently large sample for this small effect (which is a major limitation, please see section General Discussion) to become statistically significant. Please note that the study is preregistered and other than the sample size, we did not deviate from it.

^2^For exploratory purposes only (and not further analysed in the current paper), prosocialness was measured and participants were asked which version of the questionnaire they selected and why they did so, to see whether they were aware of the nudge.

^3^Only Hypotheses 1 and 2 were preregistered as confirmatory hypotheses.

^4^Preference for consistency was also measured. However, this variable was only measured for exploratory purposes and not further analysed.

^5^Although we found a mediating effect of attitude towards taking longer questionnaires on the effect of QC1 on QC2, we did not find a direct mediating effect of attitude towards taking longer questionnaires (*B* = 0.0084, SE = 0.0698, 95% CI [−0.1284, 0.1516]) and prosocial identity (*B* = 0.0042, SE = 0.0081, 95% CI [−0.0089, 0.0240]) on the effect of condition on QC2.

^6^Data would initially be analysed by using a Repeated Measures ANOVA. However, after careful consideration, we decided that such an analysis would not be best given the main question regarding the difference in food choice on the 2nd day. Therefore, we chose similar approaches to analysing the data as in Experiments 1 and 2.

^7^Although we found a mediating effect of attitude towards choosing healthier food products on the effect of NFC1 on NFC2, we did not find a direct mediating effect of attitude towards choosing healthier food products (*B* = −0.0017, SE = 0.0009, 95% CI [−0.0038, −0.0002]) on the effect of the condition on NFC2.
